# Complete Genome Sequence of a Vancomycin-Resistant Sequence Type 203 Enterococcus faecium Strain with *vanA* Belonging to Complex Type 859

**DOI:** 10.1128/MRA.00815-18

**Published:** 2018-07-19

**Authors:** Maria Hoffmann, Kuan Yao, Marc Allard, Maria Sanchez, Leif P. Andersen, Henrik Hasman, Anette M. Hammerum

**Affiliations:** a U.S. Food and Drug Administration, Center for Food Safety and Applied Nutrition, College Park, Maryland, USA; b Department of Clinical Microbiology, Rigshospitalet, Copenhagen, Denmark; c Department of Bacteria, Parasites and Fungi, Statens Serum Institut, Copenhagen, Denmark; University of Maryland School of Medicine

## Abstract

In 2014, the first vancomycin-resistant (encoded by *vanA*) Enterococcus faecium isolate belonging to sequence type 203 (ST203) and complex type 859 (CT859) was detected in Denmark. In 2016, 64% of the Danish clinical *vanA* E. faecium isolates belonged to ST203 and CT859. Using Pacific Biosciences (PacBio) RS II sequencing, we describe the genome of ST203 CT859 *vanA* E. faecium.

## Abstract (precis)

In 2014, the first vancomycin-resistant (encoded by *vanA*) Enterococcus faecium isolate belonging to sequence type 203 (ST203) and complex type 859 (CT859) was detected in Denmark. In 2016, 64% of the Danish clinical *vanA* E. faecium isolates belonged to ST203 and CT859.

## ANNOUNCEMENT

In Denmark, *vanA* Enterococcus faecium is most prevalent among clinical vancomycin-resistant Enterococcus (VRE) isolates (1).

Here, we present the complete genome sequence of a vancomycin-resistant Enterococcus faecium (VREfm) isolate, including its three plasmids, isolated from urine from a patient hospitalized at Rigshospitalet in Copenhagen, Denmark, in October 2014. E. faecium VRE1589 was the earliest *vanA* E. faecium isolate belonging to sequence type 203 and complex type 859 (ST203-CT859) identified among the VRE isolates submitted to Statens Serum Institut as part of the Danish national surveillance program. Comparison to the cgMLST.org database (https://cgmlst.org/ncs), previous Danish studies, and personal communications with neighboring countries with whole-genome sequencing (WGS) data available suggests that the novel complex type (CT859) emerged in October 2014 in Copenhagen and spread to the rest of Denmark, the south of Sweden, and the Faroe Islands during 2015 (1). In 2016, 64% of the clinical *vanA* E. faecium isolates detected in Denmark belonged to ST203 and CT859 (2).

Previous analyses showed that the E. faecium isolate VRE1589 belonged to ST203 and to a new complex type, CT859, based on the MLST version 1.8 Web server (3) and SeqSphere+ version 3.4.0 (Ridom GmbH, Münster, Germany [http://www.ridom.de/seqsphere/]) (3). For additional characterization of E. faecium VRE1589, we sequenced the isolate on a PacBio RS II sequencer. The genomic DNA was extracted with a DNeasy blood and tissue kit (Qiagen, Hilden, Germany) and sheared into approximately 20-kb fragments using g-TUBE (Covaris, Inc., Woburn, MA, USA). The library was prepared based on the 20-kb PacBio sample preparation protocol and sequenced using P6-C4 chemistry on three single-molecule real-time (SMRT) cells with a 240-min collection time. The continuous long reads were *de novo* assembled by the PacBio Hierarchical Genome Assembly Process 3.0 (HGAP3.0) program. Genomes were annotated using the National Center for Biotechnology Information (NCBI) Prokaryotic Genome Automated Pipeline version 2.9 (4) and on the RAST annotation server (http://rast.nmpdr.org/).

The presence of resistance genes was assessed using ResFinder version 2.1 (5), and plasmid replicons were detected using PlasmidFinder version 1.3 (5). The *vanA* gene was detected as part of Tn*1546* on a 49,542-bp plasmid (pVRE1589_p2) carrying three replicons similar to rep1, rep2, and rep7, with GenBank accession numbers AF007787, LT986680, and U01917, respectively. Besides *vanA*, three additional resistance genes were detected on pVRE1589_p2, *ant(6)-Ia*, *erm*(B), and *cat*(pC221). Furthermore, we detected three other resistance genes integrated on the chromosome, *msr*(C), *tet*(M), and *dfrG*. The isolate also contained a 217,014-bp plasmid (pVRE1589_p1) with a repUS15 replicon (accession number CP003586) and a 1,017-bp plasmid (pVRE1589_p3) with a rep14 replicon (GenBank accession number EFU01917); none of these plasmids contained any known resistance genes.

The origin of the ST203-CT859 *vanA* E. faecium isolate is unknown. Further studies might show why this complex type was so successful and where it originated.

### Data availability.

This complete genome project has been deposited at GenBank under accession numbers CP020484 to CP020487.
